# Management of proximal penile prosthetic cylindrical complications: a novel direct crural approach

**DOI:** 10.1186/s12610-020-00115-3

**Published:** 2020-11-03

**Authors:** Ahmed S. Zugail, Maher Abdessater, Abdulmajeed Althobity, Johnny Boustany, Mabel Nuernberg, Abdalla Alhammadi, Sébastien Beley

**Affiliations:** 1 Department of Urology, La Croix Saint Simon Hospital, Paris, France; 2 Department of Urology, Faculty of Medicine in Rabigh, King Abdulaziz University, Jeddah, Saudi Arabia; 3 Department of Urology, Sorbonne Université, APHP, Pitié Salpêtrière, 83 bvd Hopital, 75013 Paris, France; 4 Faculty of Medicine, Sorbonne University, Paris, France; 5 Department of Urology, La Pitié Salpêtrière University Hospital, Paris, France; 6 Department of Urology, Henri Mondor University Hospital, Créteil, France

**Keywords:** Voie d’abord crurale, Dysfonction érectile, Implant pénien, Prothèse pénienne, Complications prothétiques, Crural approach, Erectile dysfunction, Penile implantation, Penile prosthesis, Prosthesis failure

## Abstract

**Introduction:**

Patients with proximal penile prosthetic cylindrical complications (PPPCC) can be treated with a direct crural technique without using the original traditional approach. In this article we present our novel direct crural approach for management of patients with PPPCC.

**Materials and methods:**

Between 2014 and 2019, data were retrospectively collected from 13 patients who underwent surgical revision using our novel direct crural approach for PPPCC. The procedure commences with identification of the affected zone. The patient is in a low lithotomy position. A 2-centimeter longitudinal incision is made directly over the affected site. Dissection is carried down through Colles’ fascia, followed by a longitudinal incision through the tunica albuginea at the proximal part of the affected cylinder. Via the incision we can deliver out the cylinder and manage its problem.

**Results:**

Mean operative time was 40 min. No intra or post-operative complications were reported. All patients (Mean age = 57) were discharged on the same day. Postoperative follow-up found correction of all existing deformities at month 1, 3 and 6. All patients were satisfied and reported less pain and faster recovery than the first procedure.

**Conclusion:**

Our technique, which can be used for all types of penile prosthesis, is both feasible and safe. It may simplify PPPCC revision by avoiding adhesions below the original incision, without jeopardizing the already implanted materials or the urethra. It may also improve patients’ safety and satisfaction, by reducing iatrogenic injury and post-operative recovery time.

## Resume

**Introduction:**

Les complications concernant les cylindres proximaux des prothèses péniennes peuvent être traitées par un abord crural sans avoir recours à l’incision péno-scrotale classique. Dans cet article, on décrit une nouvelle voie d’abord crurale directe pour la prise en charge des complications prothétiques proximale.

**Matériels and Méthodes:**

Il s’agit d’une étude rétrospective, entre 2014 et 2019, sur 13 patients ayant eu une correction chirurgicale par voie d’abord crurale, suite à des complications prothétiques cylindriques proximales. La zone affectée est. identifiée avant le début de l’opération. En position de lithotomie, une incision longitudinale de 2 cm est. réalisée directement au niveau de la zone atteinte. La dissection se fait à travers le fascia de Colles, suivie d’une incision longitudinale de la tunique albuginée à la partie proximale du cylindre atteint. A travers cette incision, le cylindre peut être retiré et la gestion de son problème sera aisée.

**Résultats:**

Le temps opératoire moyen était de 40 min. Aucune complication durant ou après l’opération n’a été rapportée. Tous les patients (âge moyen = 57 ans) sont sortis de l’hôpital le même jour. Le suivi postopératoire a révélé une correction de toutes les déformations existantes après 1, 3 et 6 mois. Tous les patients étaient satisfaits et ont rapporté moins de douleur et une durée de convalescence plus courte qu’après la première procédure.

**Conclusion:**

Notre technique chirurgicale, applicable pour tout type de prothèses péniennes, est. à la fois faisable et sans risque majeur. Elle simplifie la reprise chirurgicale des complications de prothèses péniennes proximales sans avoir recours à l’incision initiale, ce qui évite les adhérences et de mettre en danger le matériel implanté et l’urètre. Elle peut également améliorer la sécurité et la satisfaction des patients en réduisant les blessures iatrogènes et le temps de récupération postopératoire.

## Introduction

Penile prosthesis technology and surgical procedures have been evolving since the early 1970’s with continual improvements in surgical outcomes. Worldwide, penile prosthesis is considered a gold standard treatment for men with organic erectile dysfunction who have failed less invasive managements and are motivated to pursue treatment and continued sexual activity [1]. Currently, the European Association of Urology indicates inflatable penile prosthesis in case of inadequate treatment outcome after phospodiesterase 5 inhibitor, topical/intraurethral alprotadil, vacuum device, low intensity shock wave treatment, and intracavernosal injections for erectile dysfunction [2]. While penile prosthesis is largely successful with low infection rates and high patient satisfaction rates, a small number of patients experience surgical or mechanical complications. Urologists performing penile prosthesis operations must be familiar with their complications, revision procedures, reconstruction techniques and salvage methods. While recent data have shown satisfactory operative rates for primary implantation of 96% at 5 years and 60% at 15 years [3], refining the surgical technique used for managing penile prosthetic cylindrical complications may further improve patient safety and satisfaction.

Simple and applicable for all types of penile prosthesis, a direct crural approach may be an effective technique for managing proximal penile prosthetic cylindrical complications while improving patient safety.

The aim of this article is to present our novel surgical technique using a direct crural approach to gain access to the proximal corpora i.e. crus of the penis *pl. crura* and repare cylinder complications, avoiding the original penoscrotal or infrapubic incision.

## Methods and materials

Data were retrospectively collected from all 13 patients who underwent consecutively our novel direct crural approach for proximal penile prosthesis cylindrical complication revision in our Urology Center in central Paris, between 2014 and 2019. Institutional review board approval was obtained before applying our novel technique and written informed consent was obtained from all patients. This article follows the TIDieR (Template for Intervention Description and Replication) reporting guidelines for medical interventions [4].

Two Urologists, one senior and one fellow, both operated all 13 patients in our Urology Center. The procedure was standardized then minimally adapted to each patient’s specified anatomy and prosthesis. The procedure was not modified during the course of study. Intervention adherence and fidelity was strong.

The direct crural approach was used for surgical revision when one cylinder was undersized or oversized. The technique was not a replacement for glansplasty in patients with hypermobile glans (frequent with Supersonic transporter (SST) deformities, two undersized cylinders or inadequate glans support).

No patient had proximal cylinder complications during the initial implant. No imaging was necessary before the surgery. Postoperative follow-up clinical examination was conducted by one of the two Urologists at month 1, 3 and 6. Complications were evaluated by clinical exam and all proximal cylinder complications were noted during follow-up.

## Surgical technique

**Fig. 1:**
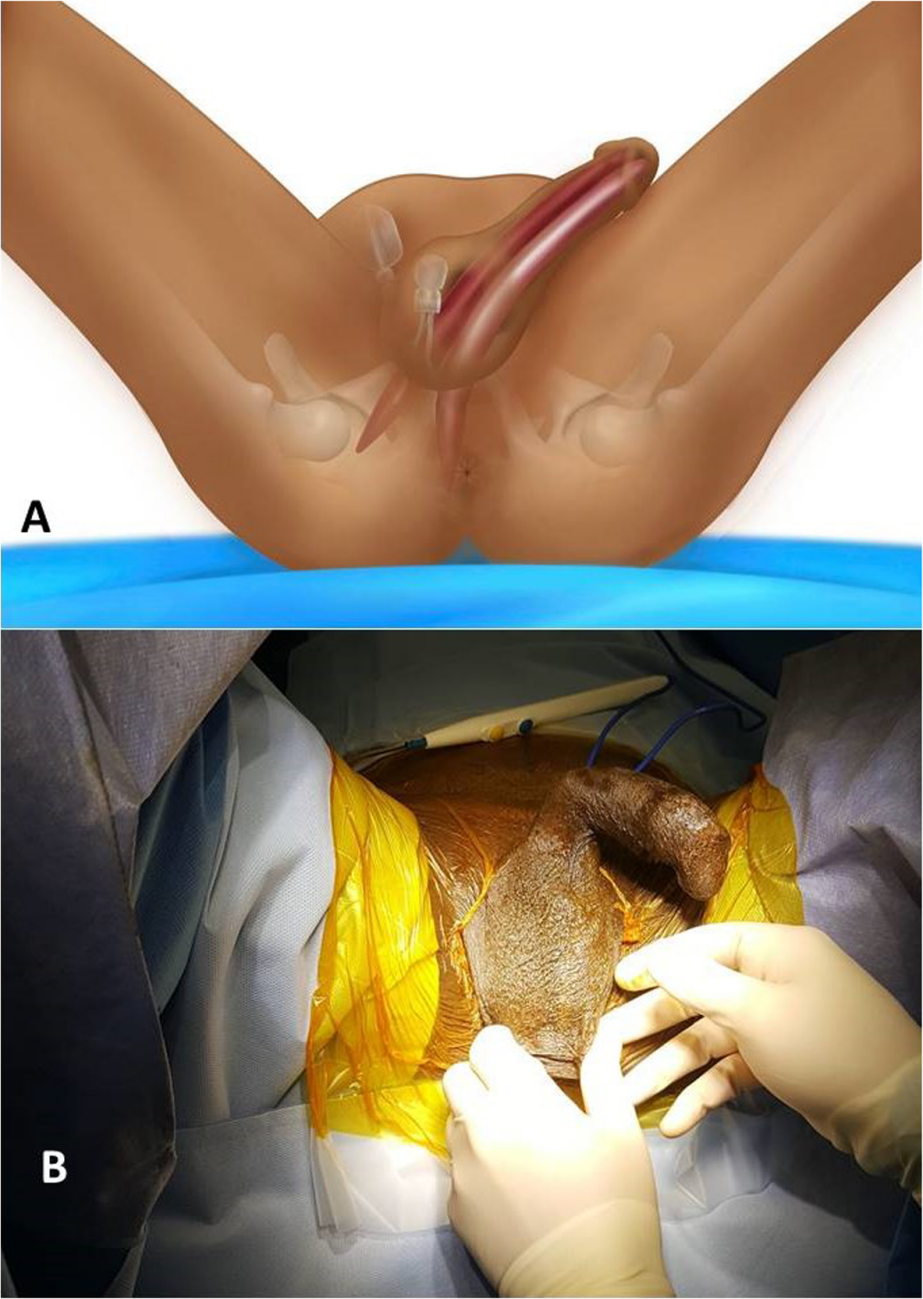
Artwork (**a)** and photo (**b)** showing the lithotomy position

**Fig. 2:**
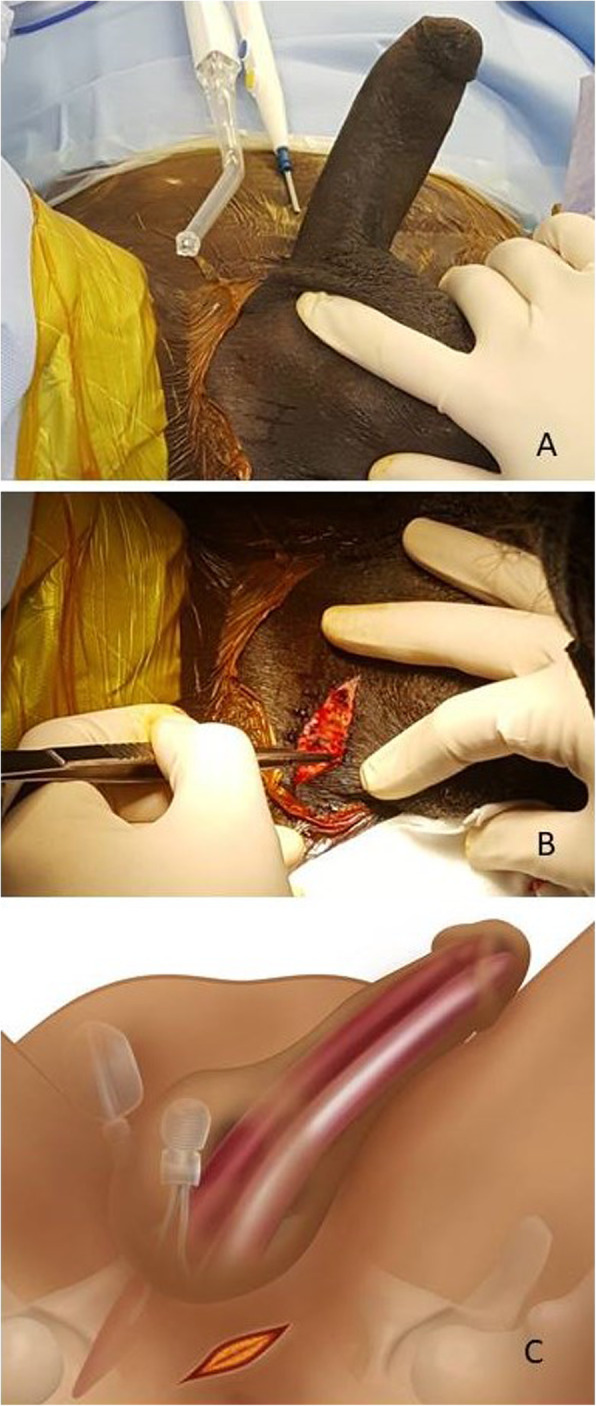
The incision of our novel technique. **a** Incisional line drawn by a sterile surgical marker and the actual longitudinal incision of 2-cm over the affected cylinder **(b)**. **c** Artwork revealing the underlying anatomical structures around the incision

**Fig. 3:**
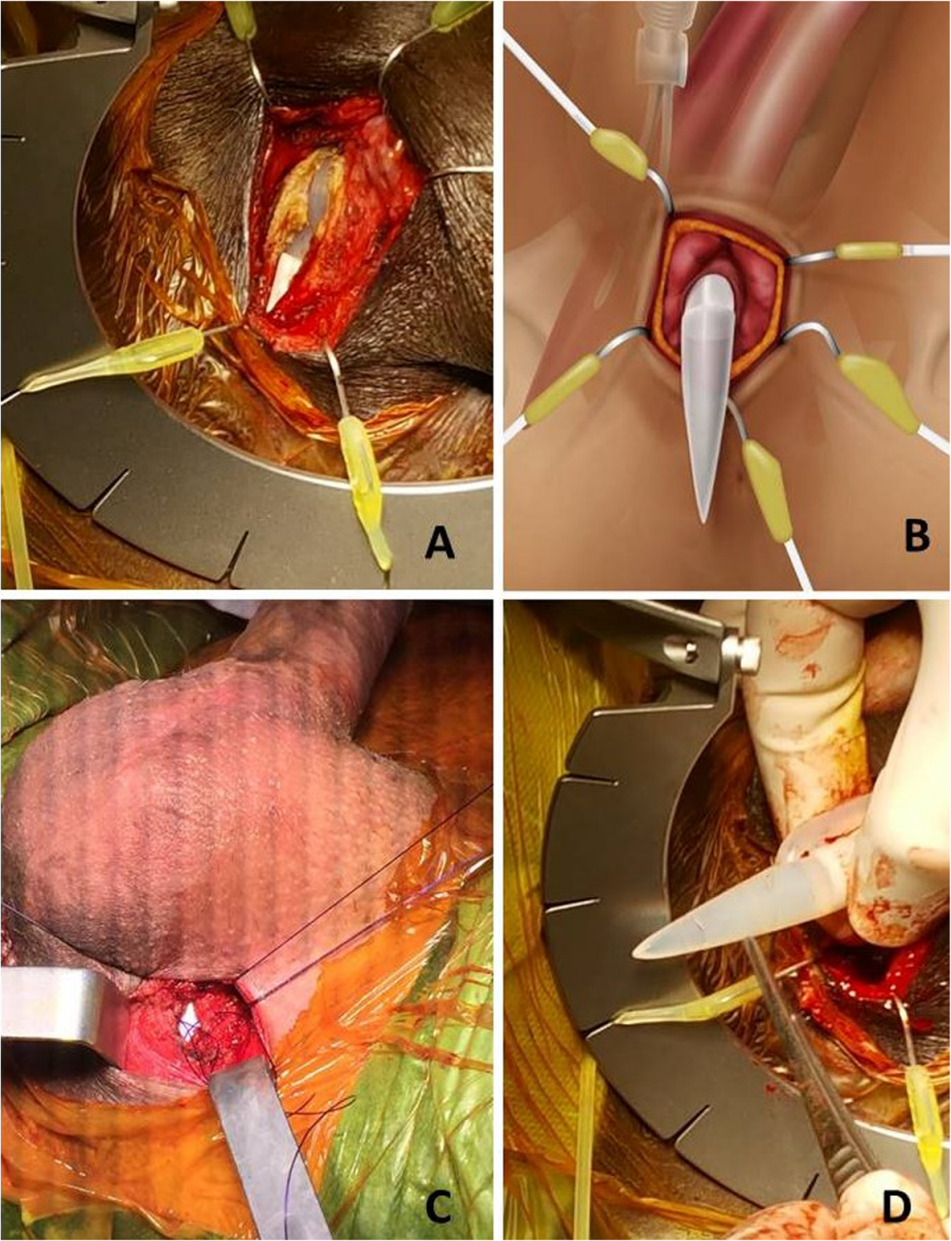
The steps of the surgical technique. **a** A photo showing the Scotts retractor in place exposing the incision of tunica albuginea with the help of multiple large blunt skin hooks. **b** Artwork revealing the underlying anatomical structures with the hooks in place. **c** A photo showing a stay 3–0 monofilament absorbable suture on each side of corporotomy that reveals the cylinder (Whitish zone). **d** The proximal end of the cylinder is easily delivered manually in this photo

Patients receive general, spinal or local anesthesia and are placed in a low lithotomy position (Fig. 1). Skin is shaved and prepped for 10 min with an iodine soapy scrub. Then, an alcoholic-iodine solution is swabbed twice followed by sterile draping using an antimicrobial incise drape to cover the area of scrotum and perineum while keeping the anus out of the surgical field. An intravenous dose of a prophylactic antibiotic is administered. The procedure commences with identification of the affected site and the nature of the deformity. A 2-centimeter longitudinal incision is made over the affected site (crus of the penis) (Fig. 2). The dissection is carried down through Colles’ fascia. A Scott’s retractor is used and large blunt skin hooks are attached to expose the corpora and facilitate further dissection. Then, a longitudinal incision through the tunica albuginea is made at the proximal part of the affected cylinder (Fig. 3a-b). A monofilament absorbable suture is placed on each side of the corporotomy as a stay suture (Fig. 3c). From this incision we can deliver out the cylinder (Fig. 3d) and manage the existing prosthetic cylinder. Previously oversized malleable or hydraulic cylinders are partially or entirely removed. Undersized cylinders are corrected either by replacing the rear tip extender (RTE) with a longer one or simply by adding one if no RTE was initially used. This incision also allows us to repair proximal cross-over and to re-position a proximally migrated cylinder or RTE back into the corpora. Then, the tunica albuginea is sealed carefully by a running and tight 3-0 monofilament absorbable stitches. The fascia and skin are tightened by 3-0 absorbable running sutures.

Patients are discharged the same day of the intervention in accordance with the Chung criteria and with appropriate pain management prescription. They are advised to take a full rest for 1 week and avoid bicycle riding or similar activities. In order to ensure adequate healing time of the tunica albuginea, patients are allowed to resume using their prosthesis 6 weeks after the surgery. The wounds are cleaned and dressed daily by a nurse using normal saline until full healing. Wound examination is conducted at all post-operative follow-up visits by the two Urologists.

## Results

Data were retrospectively collected from 13 patients who underwent our novel direct crural technique between October 2015 and March 2019. The mean age was 57 years, and ranged from 48 to 69 years. The mean body mass index (BMI) was 25 kg/m^2^ and ranged from 20 to 31 kg/m^2^. Revision procedure indications included glans bowing (SST deformity) in 6/13 (46%), proximal erosions in 4/13 (31%) and oversized cylinders with an S-shaped deformity causing pain in 3/13 (23%). No patient had previous undergone complication revision, artificial urinary sphincter insertion, or radiotherapy. Indications for the initial implant placement included vasculogenic erectile dysfunction in 6/13 (46%) and post-prostatectomy erectile dysfunction in 7/13 (54%). The Initial incision was infrapubic in 8/13 (61%) and penoscrotal in 5/13 (39%). The mean interval between initial implant placement and revision was 3 months. No patient had proximal cylinder crossovers upon intra-operative examination. Penile prosthesis model types included Titan® Touch in 10/15 (77%) and Genesis® Malleable Penile Prosthesis in 3/13 (23%) (both Coloplast Group, Humlebaek, Denmark). Prothesis length ranged from 16 to 22 cm, mean lenght was 19.7 cm. Penile prosthesis and penis length difference ranged from 1 to 3 cm mean difference was 1.5 cm.

All operations were successful with no difficulties encountered. Surgery duration ranged from 28 to 50 min, mean duration was 40 min. All 13 patients reported full satisfaction after the surgery based on the satisfaction domain of the international index of erectile function (IIEF); this domain represents the sum of the responses to questions 7, 8, 13, and 14, including intercourse satisfaction, intercourse enjoyment, overall satisfaction with sex, and satisfaction with the sexual partner. The maximum score of this domain is 20. All the patients had a score of more than 15. The patients also reported less post-operative pain and faster recovery as compared to the first procedure. All SST and S-deformities were resolved.

No complications were reported during the procedure, notably there were no iatrogenic events (urethral injuries, damage to the scrotal pump or tubing of inflatable penile prosthesis). No excessive bleeding (> 100 mL) was reported. No infections, hematomas or wound complications were reported during the median follow-up period of 6 months.

## Discussion

Surgical techniques and devices for IPP implant have evolved, improving patient safety and satisfaction [5], since Scott et al. described the first inflatable penile prosthesis (IPP) implantation in 1973 [6]. Complications occur most frequently in patients with diabetes, spinal cord injury or immunosuppression [7]. Revision procedures after IPP implantation are most frequently due to cosmetic or erosive etiologies as opposed to mechanical issues [8]. As increasing numbers of patients with erectile dysfunction undergo penile prosthetic implantation, unusual complications are being reported. This highlights the need for new, simpler and safer revision techniques [9].

Accessing the corpora through direct scrotal incision may be beneficial as it avoids the prior penoscrotal or high scrotal incisions and surgical difficulties due to adhesions and fibrosis from the prior intervention. However, the risk of iatrogenic injury due to the direct scrotal approach is non-negligible. The scrotal pump or tubing may be threatened. The biofilm capsule may be violated during dissection, possibly leading to post-operative infection. Scrotal edema or hematoma may prolong the post-operative recovery period and delay sexual activity resumption.

The direct crural approach may be an alternative surgical method useful to treat penile prosthetic cylinder complication and manage existing abnormalities. Prosthetic cylinder revision using our technique may be safe and efficient; the single crural incision provides a more superficial access to the corpora with less tissue manipulation as compared to the traditional scrotal approach. Our novel direct crural approach facilitates surgical repair and provides better surgical field exposure and thus a better anatomical perspective. The technique can be used for both inflatable and semirigid (malleable) penile prostheses. It is useful in revising proximal penile prosthetic cylindrical complications such as oversized cylinders causing S-shaped deformities, undersized cylinders causing incompletely dilated corpora and Glans Bowing (Penile supersonic transporter [SST] deformities), and proximal cylinder erosions beneath the skin and proximal cylinder crossovers. Both iatrogenic injury and post-operative recovery time may be reduced as the approach avoids adhesions below the original incision, without jeopardizing the already implanted materials or the urethra. To note that it is widely demonstrated that revision procedures of penile prosthesis are at higher risk of complications. This is due first to corporal fibrosis or infection. In this context, studies indicate a complications rate (especially infectious ones) ranging from 8 to 12% [10–13].

The reduction of surgical time is possibly one of the main reasons for success of our technique, with the consequent reduction of the risk of infection. The prosthetic surgeon will always prefer the simple, fast and clean procedure.

This direct crural approach has several limitations. First, it is not appropriate for numerous penile prosthetic cylinder complications including distal corporal erosions beneath the skin or glans deformities that need plication. Thus it could not be a good option in case of cylinder crossing due to the difficulty in controlling the rest of the corpus cavernosum It almost invariably requires bilateral access, regardless of the type of prosthesis, being a problem of incorrect implant size. Second, this approach allows only a limited exploration of the distal corpora, an essential step in achieving successful outcomes. It is not adapted to patients requiring implantation of a new device and it should be further explored in patients presenting prosthetic cylinder complications requiring a bilateral approach. Finally, we would highlight that here, we are only considering a very small series from a non-comparative study which preclude conclusions on a large scale.

## Conclusion

Our novel direct crural approach is safe, simple and effective in managing certain penile prosthetic cylindrical complications requiring access to the proximal cylinder. This approach can be used for both inflatable and semi rigid (malleable) penile prostheses. It avoids adhesions below the original incision, without jeopardizing the already implanted materials or the urethra. However, further exploration of this technique is required before it may be used as a standard surgical method.

## Data Availability

Not applicable.

## Abbreviations

BMI: Body mass index
IIEF: International index of erectile function
PPPCC: Proximal penile prosthetic cylindrical complications
RTE: Rear tip extender
SST: Supersonic transporter
TIDieR: Template for intervention description and replication
